# Multiple-locus variable-number tandem repeat analysis for molecular typing of *Aspergillus fumigatus*

**DOI:** 10.1186/1471-2180-10-315

**Published:** 2010-12-08

**Authors:** Simon Thierry, Dongying Wang, Pascal Arné, Manjula Deville, Barbara De Bruin, Adélaïde Nieguitsila, Christine Pourcel, Karine Laroucau, René Chermette, Weiyi Huang, Françoise Botterel, Jacques Guillot

**Affiliations:** 1 ANSES, UMR BIPAR, Ecopham, Agence Nationale de Sécurité Sanitaire, Maisons-Alfort, France; 2 Parasitology department, College of Animal Science and Technology, Guangxi University, Nanning, China; 3 ENVA, UMR BIPAR, Ecopham, Ecole nationale vétérinaire d'Alfort, Maisons-Alfort, France; 4 Université Paris Sud 11, CNRS, UMR 8621, Institut de Génétique et Microbiologie, Orsay 91405, France; 5 ANSES, Bacterial Zoonoses Unit, Maisons-Alfort, France; 6 UPE, UMR BIPAR, Ecopham, Faculté de Médecine de Créteil, France

## Abstract

**Background:**

Multiple-locus variable-number tandem repeat (VNTR) analysis (MLVA) is a prominent subtyping method to resolve closely related microbial isolates to provide information for establishing genetic patterns among isolates and to investigate disease outbreaks. The usefulness of MLVA was recently demonstrated for the avian major pathogen *Chlamydophila psittaci*. In the present study, we developed a similar method for another pathogen of birds: the filamentous fungus *Aspergillus fumigatus*.

**Results:**

We selected 10 VNTR markers located on 4 different chromosomes (1, 5, 6 and 8) of *A. fumigatus*. These markers were tested with 57 unrelated isolates from different hosts or their environment (53 isolates from avian species in France, China or Morocco, 3 isolates from humans collected at CHU Henri Mondor hospital in France and the reference strain CBS 144.89). The Simpson index for individual markers ranged from 0.5771 to 0.8530. A combined loci index calculated with all the markers yielded an index of 0.9994. In a second step, the panel of 10 markers was used in different epidemiological situations and tested on 277 isolates, including 62 isolates from birds in Guangxi province in China, 95 isolates collected in two duck farms in France and 120 environmental isolates from a turkey hatchery in France. A database was created with the results of the present study http://minisatellites.u-psud.fr/MLVAnet/. Three major clusters of isolates were defined by using the graphing algorithm termed Minimum Spanning Tree (MST). The first cluster comprised most of the avian isolates collected in the two duck farms in France, the second cluster comprised most of the avian isolates collected in poultry farms in China and the third one comprised most of the isolates collected in the turkey hatchery in France.

**Conclusions:**

MLVA displayed excellent discriminatory power. The method showed a good reproducibility. MST analysis revealed an interesting clustering with a clear separation between isolates according to their geographic origin rather than their respective hosts.

## Background

The filamentous fungus *Aspergillus fumigatus *thrives on decaying vegetation and organic debris. It releases large amounts of asexual spores (conidia), which are dispersed by air. As a result of this ubiquitous presence, people and animals are constantly exposed to *A. fumigatus *conidia. In humans, conidia can colonize the respiratory tract, causing pulmonary infections including bronchopulmonary aspergillosis, aspergilloma and invasive aspergillosis. In birds, respiratory aspergillosis is considered as a major cause of morbidity and mortality. Aspergillosis is frequently reported in turkey poults, in quails, in marine birds that are brought into rehabilitation, in captive raptors, and in penguins being maintained in zoological parks [1-3].

The Multiple Locus Variable-number tandem-repeat Analysis (MLVA) is based on polymorphism of tandemly repeated genomic sequences called VNTR (Variable-Number Tandem-Repeats). VNTRs are classically separated into microsatellites (up to 8 bp) and minisatellites (9 bp and more) [4]. The MLVA technique has been used for the genotyping of many bacterial pathogens [5-12] as well as the opportunistic yeast *Candida glabrata *[13]. For these pathogens, MLVA technique allowed to resolve closely related microbial isolates for investigation of disease outbreaks and provided information for establishing phylogenetic patterns among isolates. The MLVA technique can be performed with simple electrophoretic equipment. The usefulness of MLVA was recently demonstrated for the avian major pathogen *Chlamydophila psittaci *[5].

The objective of the present study was to develop a new typing method based on the detection of VNTRs in the filamentous fungus *A. fumigatus*, another major avian pathogen. All putative VNTR markers were screened on the whole genome of *A. fumigatus *strain Af293. Ten markers were finally selected and used for the typing of a large number of isolates from poultry and their environment in France and China.

## Methods

### Strain collection

In order to develop a MLVA scheme and choose discriminant VNTR markers, a total number of 57 isolates was selected from our laboratory collection. These isolates were considered as geographically or temporally unrelated. The isolates were collected from tissues or from pharyngeal swabs: (i) 49 isolates from different animal species with lesions of aspergillosis in different places in France (n = 48) or Morocco (n = 1); (ii) 3 isolates collected from human cases of aspergillosis at one hospital in Ile-de-France region, France; (iii) 2 isolates collected from healthy birds in 2 poultry farms in France; (iv) 2 isolates from healthy birds in chicken and duck farms in Guangxi province, China; (v) the reference strain CBS 144.89 (Table 1).

**Table 1:** Origin and period of collection for 57 unrelated isolates of Aspergillus fumigatus examined in the present study

Isolates no	Hosts	Period of collection	Geographic origin
S1-S15, S24, S25, S28-S35, S38, S46, S48, S49, S50, S51, S54, S55	Ducks (*Anas platyrhynchos*), pulmonary aspergillosis	10/2007-04/2008	Poitou-Charentes, France

S17, S18	Pigeons (*Columba livia*), pulmonary aspergillosis	11/2007	Ile de France, France

S19, S20, S22, S23, S26, S36, S42, S44, S52, S53, S56	Turkey (*Meleagris gallopavo*), pulmonary aspergillosis	11/2007-04/2008	Poitou-Charentes, France

S40, S41	Pheasant (*Phasianus colchicus*), pulmonary aspergillosis	01/2008	Poitou-Charentes, France

E19, E20	Ducks (*Anas platyrhynchos*), asymptomatic carriage in pharynx	01/2008-04/2008	Sarthe, France

D3	Chicken (*Gallus gallus*), asymptomatic carriage in pharynx	02/2008-03/2008	Guangxi province, China

D42	Duck (*Anas platyrhynchos*), pulmonary aspergillosis	02/2008-03/2008	Guangxi province, China

V04M02253	Bustard (*Chlamydotis undulata*), asymptomatic carriage in trachea	01/2008-04/2008	Morocco

H50, H71, H100	Patients, pulmonary aspergillosis	12/2005-04/2008	Ile de France, France

CBS 144.89	Patient, invasive aspergillosis	-	France

To test the MLVA technique, we selected a second group of 277 isolates which represented 5 distinct epidemiological situations: (i) 52 isolates collected during an epidemiological survey conducted in the same duck farm in 2008 in Sarthe department, France; (ii) 43 isolates collected during an epidemiological survey conducted in another single duck farm in 2008 in Sarthe department, France; (iii) 48 isolates collected during an epidemiological survey in a chicken farm in 2008 in Guangxi province, China; (iv) 14 isolates collected during an epidemiological survey in a duck farm in 2008 in Guangxi province, China; (v) 120 environmental isolates collected in 2009 in a turkey hatchery in Maine-et-Loire department, France (Table 2).

**Table 2:** Origin and period of collection for 277 epidemiologically related isolates of Aspergillus fumigatus

Isolates no	Samples	Period of collection	Geographic origin
E1-2, E5, E8-9, E10, E13-19, E21-23, E26, E29, E30, E32-34, E36-38, E40-45, E51-53, E57, E59-64, E69-70, E72, E74-75, E79, E82-83, E85-86, E90	Pharyngeal swabs from ducks (*Anas platyrhynchos)*	01/2008-04/2008	Farm A in Sarthe, France

E3-4, E6-7, E11-12, E20, E24-25, E27-28, E31, E35, E39, E46-50, E54-56, E58, E65-68, E71, E73, E76-78, E80-81, E84, E87-89, E91-95	Pharyngeal swabs from ducks (*Anas platyrhynchos)*	01/2008-04/2008	Farm B in Sarthe, France

D1-40, D59-66	Pharyngeal swabs from chickens (*Gallus gallus*)	02/2008-03/2008	Farm C in Guangxi province, China

D41-54	Pharyngeal swabs from ducks (*Anas platyrhynchos*)	02/2008-03/2008	Farm D in Guangxi province, China

G1-120	Air samples from a turkey hatchery	11/2008-03/2009	Hatchery in Maine et Loire, France

To test the specificity of the MLVA technique, isolates from other *Aspergillus *species (*A. lentulus *CBS 117885*, A. flavus *environmental isolate, *A. nidulans *CBS 589.65 and *A. niger *CBS 733.88 and environmental isolate) were also included.

*Aspergillus *isolates were microscopically identified after cultivation on Malt Agar plates at 37°C until conidia formation. For 95 randomly selected isolates, the species identification was confirmed by amplification and sequencing of partial β-Tubulin gene using primer set βtub1-βtub2 [14,15].

### DNA isolation

From each isolate, conidia were collected from the culture and transferred into a microtube for extraction. A bead mill homogenization step was used, before the lysis treatment, to facilitate the disruption of the complex fungal cell wall. Bead mill homogenization was carried out in a high-speed (5000 rpm) mini-bead beater (Mixer Mill MM301, Qiagen, Courtaboeuf, France). Lysis and DNA extraction were then performed using the Nucleospin DNA Extraction Kit (Macherey-Nagel, Germany).

### Selection of VNTR markers

The availability of the whole genome sequence of *A. fumigatus *strains (strain Af293) allowed us to search for tandem-repeat sequences in the Tandem Repeat Database of the University Paris Sud XI in Orsay http://minisatellites.u-psud.fr/GPMS/ using the Tandem Repeat Finder software [16]. In order to evaluate the polymorphism of selected tandem repeats, primers were chosen on both sides of the repeats and the 57 unrelated isolates from our laboratory collection were analyzed. PCR were performed in a total volume of 15 μl containing 1-5 ng of DNA, 1X PCR reaction buffer, 0.5 U of Taq polymerase (Takara Bio Inc, Shiga Japan), 250 μM of each deoxynucleotide triphosphate, and 0.5 μM of each flanking primer. Primers were designed using Primer Express^® ^2.0 software. The initial denaturation step at 95°C for 10 min was followed by 35 cycles consisting of denaturation at 95°C for 30 s, primer annealing at 58°C for 40 s, and elongation at 72°C for 30 s. The final extension step was at 72°C for 10 min.

**Figure 1:**
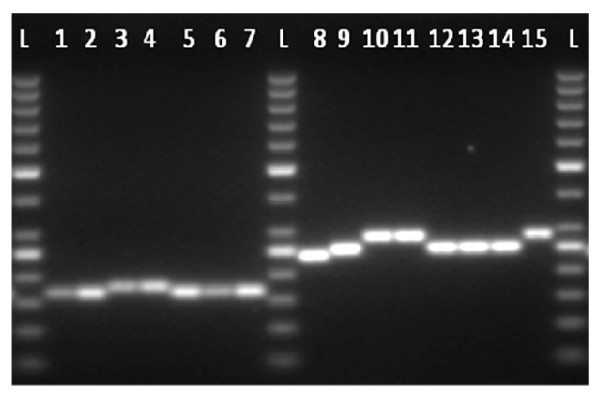
**Electrophoretic gel showing VNTR profiles**. Lanes 1 to 7 represent the amplification of 7 isolates for marker Asp_330 (11 bp repeat). Samples in lanes 1-2 and 5-7 have 3 repeats and samples in lanes 3-4 have 4 repeats. Lanes 8 to 15 represent the amplification of 8 other isolates for Asp_443 marker (18 bp repeat). Sample in lane 8 has 4 repeats, samples in lanes 9 and 12-14 have 5 repeats, samples in lanes 10-11 and 15 have 7 repeats.

Ten microliters of amplification product were loaded onto a 3% standard agarose gel. Gels stained with ethidium bromide were visualized under UV light, and photographed (Figure 1). The size marker used was a Quick-load 100-bp ladder (New England BioLabs, Ipswich UK).

### Sequencing

The alleles observed on each VNTR were sequenced to confirm the observations made on electrophoresis gel. The number of repeats was estimated from the amplicon size. The sequencing of one example of allele allowed to check whether microdeletions occurred and to evaluate the internal variation of the repeats. A total number of 70 amplicons were sequenced by Qiagen (Courtaboeuf, France) and then aligned and compared, in order to confirm the exact number of repeats.

### Stability and reproducibility

The stability of the VNTR markers was estimated by analysis of 5 distinct isolates of *A. fumigatus *subcultured 12 times in 2 months.

The reproducibility of the method was assessed by the analysis of 8 isolates in 2 different units situated in two different buildings of the Animal Health Laboratory of ANSES (Agence Nationale de Sécurité Sanitaire, Alimentation, Environnement, Travail) at Maisons-Alfort, France, and by 2 different technicians.

### Discriminatory power

The discriminatory power was calculated by using the Simpson index of diversity (*D*):

$$D=1-\frac{1}{N\left(N-1\right)}\sum_{j=1}^{s}n_{j}\left(n_{j}-1\right)$$

where N is the total number of isolates in the test population (57 unrelated isolates), s is the total number of types described, and nj is the number of isolates belonging to the j^th ^type [17]. A D value of 1.0 indicates that the typing method is able to discriminate between all isolates. A D value of 0.0 indicates that all isolates are identical.

### Clustering analysis

Amplicon size was determined with Bionumerics software package version 4.6 (Applied-Maths, Saint-Martens-Latem, Belgium). The number of repeats in each allele was derived from the amplicon size. The size of flanking sequences was subtracted from the band size and the number was divided by the repeats size. The result of this calculation corresponded to the number of repeats. Data were analyzed with Bionumerics software as a character dataset. Two different techniques were used to represent the relationships between isolates [12]: (i) a phenogram using phenetic UPGMA method (Data not shown; (ii) a graphing algorithm termed Minimum Spanning Tree (MST). The priority rule for constructing MST was set in order that the type that had the highest number of single-locus variants (SLVs) would be linked first. A cutoff value of maximum differences of 2 VNTRs out of 10 was applied to define cluster in the MST method.

## Results

### Selection of VNTR markers

The use of the Tandem Repeat Finder software allowed the detection of 77 tandem repeats with a repeat unit larger than 9 bp. Putative VNTR markers were found in the 8 chromosomes of *A. fumigatus*. For the selection of markers, 2 criteria were used: a homology of more than 90% between the different repeats and a number of repeats higher than 3. Only 10 out of these markers were polymorphic in the 57 unrelated isolates. The 10 markers were located on 4 different chromosomes (1, 5, 6 and 8). Five VNTRs were on chromosome 1 (Asp_167, Asp_202, Asp_330, Asp_443 and Asp_446). VNTRs Asp_165, Asp_252 and Asp_345 were on chromosome 5. VNTRs Asp_204 and Asp_20 were on chromosome 6 and 8, respectively. Characteristics of final VNTRs and respective primer sets are listed in Table 2.

Considering that the genome of *A. fumigatus *is haploid, we excluded isolates presenting double-bands patterns, because these patterns could be explained by a mixture of genotypes. When mixtures were suspected, different colonies were separated and subcultured. Each colony genotype was characterized by a distinct MLVA pattern (data not shown). This result proved that double-bands patterns were due to mixtures of isolates.

### Stability and reproducibility

The 60 samples (5 isolates subcultured 12 times in 2 months) used for the evaluation of stability were typed by MLVA and yielded exactly the same MLVA pattern.

The 16 samples used for the evaluation of reproducibility (8 isolates tested by 2 different technicians in 2 different laboratories) yielded exactly the same MLVA pattern.

### Discriminatory power

Simpson diversity index was first calculated for each VNTR and for the panel of 10 markers tested on the 57 unrelated isolates. The index for individual markers ranged from 0.5771 to 0.8530 (Table 3). A combined loci index calculated with all of 10 markers yielded an index of 0.9994. When the VNTR profiles of 330 isolates were considered, the combined loci index was 0.9956.

**Table 3:** Characteristics of VNTR markers for fingerprinting of Aspergillus fumigatus

VNTR markers	Primer sequences (5' to 3')	Tm (°C)	Unit repeat size (bp)	Range of repeat number	Simpson diversity index*	Marker location (non coding region or name of gene if coding)
Asp 167	TGAGATGGTTAACTTACGTAGCGC CGCTCCCACCGTTACCAAC	59	12	4-8	0.7151	Chromosome 1 (GPI anchored serine-rich protein)

Asp 202	AGGATCACTGCCCTCAACCC CCGAAATCCGCGGGA	59	12	6-14	0.8530	Chromosome 1 (c-24(28) sterol reductase)

Asp 330	ATCTGGTCGCGAAATTCCTCT TCTTCGGCCTTTTCATCCC	58	11	2-8	0.7895	Chromosome 1 (non coding)

Asp 443	AAGCTTCGTCTGGCGAAGAG GCACGTGTACGGTGTTCCTG	58	18	0-7	0.6661	Chromosome 1 (ribosome assembly protein Noc2)

Asp 446	CGATCATGTTTGCCTGAGGA CCGACAGCATCGAGCAACTA	59	21	1-4	0.5971	Chromosome 1 (non coding)

Asp 165	TGATGGGCCGCAGTCG GCACCTGCTTGTCGATTCGT	60	10	0-6	0.7296	Chromosome 5 (non coding)

Asp 252	CAGATTGGAGACACGAAGCG ACCACGGATTGCCAAGGA	58	12	2-6	0.5886	Chromosome 5 (non coding)

Asp 345	TCTCCAACCCTTCGGACG GCCGGAAGAGCATGAAGACA	58	11	1-6	0.5771	Chromosome 5 (non coding)

Asp 204	GATGCGGGAGGTGGGTC CGTCCTCACTTTTGCCTTGG	58	11	1-5	0.6128	Chromosome 6 (non coding)

Asp 20	GGGAAGAGAGGAACCGATCC CGCAGTGGGCAGTTTGAAT	58	10	0-4	0.7520	Chromosome 8 (non coding)

*Each index was calculated with the results from 57 unrelated *A. fumigatus *isolates

### Accessibility through the web

A database was created with the results of the present study http://minisatellites.u-psud.fr/MLVAnet/. On this website, it is possible to compare VNTR patterns with 300 different patterns included in the database using complete panel of markers or just a selection of them. This database also allows to build dendrograms with the query. All the possibilities provided by the website and database are explained by Grissa *et al*. [18].

### Specificity

When VNTR primer sets were tested with DNA from *Aspergillus flavus*, *A. niger *and *A. nidulans *no amplification was observed. When VNTR primer sets were tested with DNA from *Aspergillus lentulus*, a species closely related to *A. fumigatus*, amplification was obtained with 3 out of 10 markers (Asp_167, Asp_202 and Asp_330). As a consequence the combination of 10 VNTRs should be considered as specific of *A. fumigatus*.

### Clustering analysis

A total number of 330 *A. fumigatus *isolates were typed with the panel of 10 VNTRs. This analysis yielded 255 different genotypes. Only 33 genotypes were shared by two isolates or more.

UPGMA analysis did not allow a clear clustering of the isolates (data not shown). Some isolates (n = 12) were characterized by the insertion of a large sequence (about 450 bp) in VNTR Asp_20 whereas others (n = 6) had a very high number of repeats (from 10 to 17) in the VNTR Asp_202 and (from 10 to 15) in the VNTR Asp_330, exhibiting patterns which were not observed in the group of unrelated isolates (Table 3).

**Figure 2:**
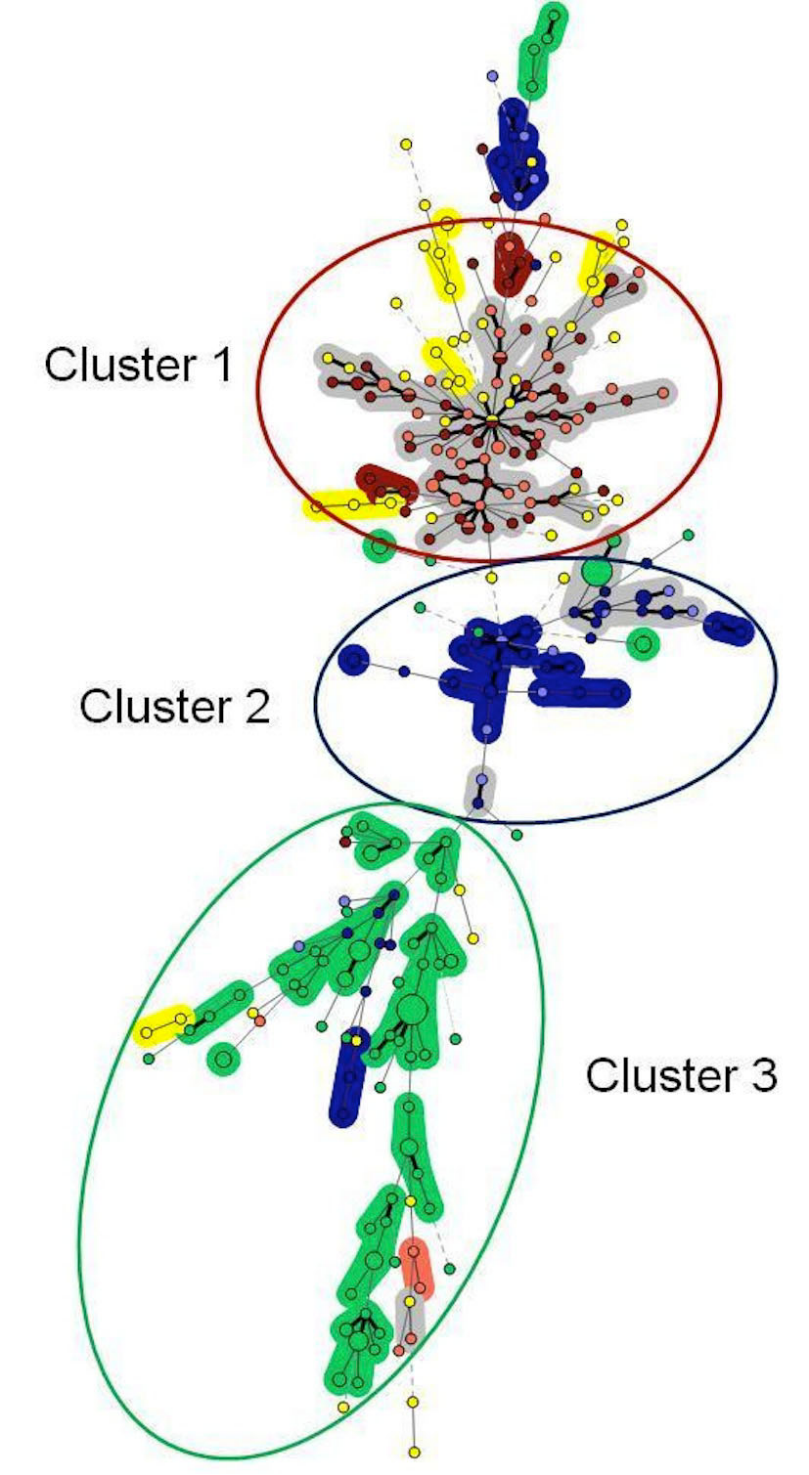
**Minimum spanning tree of 330 *A. fumigatus *isolates based on categorical analysis of 10 VNTRs**. Each circle represents a unique genotype. The diameter of each circle corresponds to the number of isolates with the same genotype. Genotypes connected by a shaded background differ by a maximum of 2 of the 10 VNTR markers and could be considered a "clonal complex". Thick connecting lines represent one marker difference; regular connecting lines represent two marker differences; thick interrupted lines represent three differences; thin interrupted lines represent four or more differences. The length of each branch is also proportional to the number of difference. Each epidemiological situation is represented by a specific colour: red for isolates collected during an epidemiological survey conducted in the same duck farm in 2007 and 2008 in Sarthe region, France; salmon pink for isolates collected during an epidemiological survey conducted in another single duck farm in 2007 and 2008 in Sarthe region, France; navy blue for isolates collected during an epidemiological survey in a chicken farm in 2008 in Guangxi province, China; light blue for isolates collected during an epidemiological survey in a duck farm in 2008 in Guangxi province, China; green for environmental isolates collected in 2009 in a turkey hatchery in Maine et Loire region, France and yellow for unrelated isolates. Large ellipses correspond to the three major clusters (with the colour of the majority of the genotypes).

The graphing algorithm termed Minimum Spanning Tree (MST) demonstrated three major clusters of isolates (Figure 2). The first cluster comprised 91 out of 95 avian isolates (95%) collected in the two duck farms in Sarthe department in France. The second cluster comprised 42 out of 62 avian isolates (70%) collected in poultry farms in Guangxi province in China and the third cluster comprised 90 out of 120 environmental isolates (75%) from the turkey hatchery in Maine-et-Loire department in France. In the dendrogram, genotypes corresponding to unrelated isolates are clearly separated.

## Discussion

Typing *A. fumigatus *isolates may help to improve the understanding of the distribution of this major pathogen in different situations and environments, including susceptible birds in poultry farms. This molecular approach may also give a deeper understanding of the colonization pattern of putative hosts. To date, it is still a matter of controversy whether certain isolates are more virulent and genetically distinct from other isolates, or whether infection by *A. fumigatus *is simply a matter of contracting infection from any environmental source.

The choice of a specific typing technique depends on the scientific questions and the equipment of the laboratory. Many different techniques have already been described for *A. fumigatus*: Random Amplified Polymorphic DNA (RAPD) [19], Restriction Enzyme Analysis (REA) [20], Restriction Fragment Length Polymorphism (RFLP) [21], Amplified Fragment Length Polymorphism (AFLP) [22], Microsatellite Length Polymorphism (MLP) [23-27] and Multilocus Sequence Typing (MLST) [28]. CSP typing is a recently developed typing strategy that involves DNA sequence typing of a repetitive region of the *A. fumigatus *AFUA_3G08990 gene coding for a Cell Surface Protein, designated the CSP locus [29,30]. All of these typing techniques were developed in order to resolve closely related isolates for the purposes of outbreak investigation in hospitals and disease surveillance in humans. RFLP (with Afut1 probe) and MLP typing methods were proved to be highly discriminant. Furthermore MLP showed high reproducibility because of the unambiguous data. For these reasons, MLP method is now considered as the gold standard for the analysis of epidemiological relationships between large amounts of *A. fumigatus *isolates over a long period of time in hospitals. Another method with high reproducibility is MLST, but the loci described so far for *A. fumigatus *are probably not discriminant enough to identify the source of an outbreak situation. The RAPD method was used in many investigations probably because it requires simple equipment and no genomic sequence information, but it suffered from limited discriminatory power and reproducibility.

In the present study, a molecular typing method for *A. fumigatus *based on the study of 10 VNTR markers with repeat size larger than 9 bp was developed and further applied to 277 isolates from birds or from the environment. The MLVA typing method proved highly discriminant with a Simpson's diversity index of 0.9994. This value was obtained with unrelated isolates from animals or humans and was exactly the same as that obtained with isolates from humans using microsatellite markers [25]. Size differences between alleles of the 10 selected VNTRs were large enough to allow efficient sizing on agarose gel. This makes the present MLVA scheme easy to implement in laboratories possessing basic molecular biology equipment. The method showed a good reproducibility, which could be increased by the production of an internal ladder (including an example of each allele amplicon size) or the use of capillary electrophoresis [31]. The MLVA was shown to be rapid and very discriminant. Performing monoplex amplifications, like in the present study, leads to more effort than using multiplex amplifications. In future development of the MLVA technique, the combination of two or more VNTR amplifications in a single reaction tube should be tested.

For the clustering analysis of VNTR profiles, we used a graphing algorithm termed minimum spanning tree (MST). This method was introduced to improve analysis of VNTR profiles [15]. Similar to maximum-parsimony phylogenetic tree reconstruction methods, MST constructs a tree that connects all the genetic profiles in such a way that the summed genetic distance of all branches is minimized. The differences in mathematical approach between MST and UPGMA methods may account for the changes in isolates clustering. Thus, MST allowed to group *A. fumigatus *isolates which were unclustered with UPGMA. A first cluster included most of the isolates from birds in France whereas the second included most of the isolates from birds in China (Figure 2). The third cluster included most of the environmental isolates collected in a hatchery in France. As a consequence, MST results clearly reflected the geographic origin of the isolates. However, the clustering did not allow the separation of isolates collected from birds living in two different farms in the same department (in France) or province in China. This suggests that geographic clustering occurs at the scale of large areas. The distance between the two farms in Sarthe department in France or in Guangxi province was 20 km and 30 km, respectively. The mean distance between the two farms in Sarthe department and the hatchery in Maine-et-Loire was 120 km. To confirm the geographic clustering and evaluate the minimum size of geographic clusters, additional samples from other origins should be included. We should also collect environmental isolates near the poultry farms in Sarthe department or Guangxi province and avian isolates near the hatchery in Maine-et-Loire department. Geographic clustering of *A. fumigatus *isolates using repeat sequence analysis with the CSP method, was suggested by Balajee in 2007 [29]. Recently, another study using the AFLP method showed a geographic structuration of *A. fumigatus *isolates [32].

## Conclusions

The present study allowed to describe 10 VNTR markers, applicable in the typing of the major fungal pathogen *Aspergillus fumigatus*. The loci in this VNTR assay were highly discriminating and stable over time. The typing method could be used for molecular epidemiological studies of *A. fumigatus *in different situations including avian farms and hospitals where outbreaks of invasive aspergillosis may occur. Furthermore, data obtained by the present method could be easily shared in a web database

## Authors' contributions

ST, PA, CP, RC, WH and JG participated in the design of the study, participated in the phylogenetic analysis and draft the manuscript. ST, DW, MD, BDB and AN participated in the molecular studies. KL helped in the collection of isolates from poultry farms in France and participated in the design of the study. DW collected isolates from poultry farms in China. FB participated in draft of the manuscript. All the authors read and approved the final manuscript.
